# The critical role of the iron–sulfur cluster and CTC components in DOG-1/BRIP1 function in *Caenorhabditis elegans*

**DOI:** 10.1093/nar/gkae617

**Published:** 2024-07-16

**Authors:** Xiao Li, Ivette Maria Menendez Perdomo, Victoria Rodrigues Alves Barbosa, Catherine Diao, Maja Tarailo-Graovac

**Affiliations:** Department of Biochemistry and Molecular Biology, Cumming School of Medicine, University of Calgary, Calgary, Alberta, T2N 4N1, Canada; Department of Medical Genetics, Cumming School of Medicine, University of Calgary, Calgary, Alberta, T2N 4N1, Canada; Alberta Children’s Hospital Research Institute, University of Calgary, Calgary, Alberta, T2N 4N1, Canada; Department of Biochemistry and Molecular Biology, Cumming School of Medicine, University of Calgary, Calgary, Alberta, T2N 4N1, Canada; Department of Medical Genetics, Cumming School of Medicine, University of Calgary, Calgary, Alberta, T2N 4N1, Canada; Alberta Children’s Hospital Research Institute, University of Calgary, Calgary, Alberta, T2N 4N1, Canada; Department of Biochemistry and Molecular Biology, Cumming School of Medicine, University of Calgary, Calgary, Alberta, T2N 4N1, Canada; Department of Medical Genetics, Cumming School of Medicine, University of Calgary, Calgary, Alberta, T2N 4N1, Canada; Alberta Children’s Hospital Research Institute, University of Calgary, Calgary, Alberta, T2N 4N1, Canada; Department of Biochemistry and Molecular Biology, Cumming School of Medicine, University of Calgary, Calgary, Alberta, T2N 4N1, Canada; Department of Medical Genetics, Cumming School of Medicine, University of Calgary, Calgary, Alberta, T2N 4N1, Canada; Alberta Children’s Hospital Research Institute, University of Calgary, Calgary, Alberta, T2N 4N1, Canada; Department of Biochemistry and Molecular Biology, Cumming School of Medicine, University of Calgary, Calgary, Alberta, T2N 4N1, Canada; Department of Medical Genetics, Cumming School of Medicine, University of Calgary, Calgary, Alberta, T2N 4N1, Canada; Alberta Children’s Hospital Research Institute, University of Calgary, Calgary, Alberta, T2N 4N1, Canada

## Abstract

FANCJ/BRIP1, initially identified as DOG-1 (Deletions Of G-rich DNA) in *Caenorhabditis elegans*, plays a critical role in genome integrity by facilitating DNA interstrand cross-link repair and resolving G-quadruplex structures. Its function is tightly linked to a conserved [4Fe–4S] cluster-binding motif, mutations of which contribute to Fanconi anemia and various cancers. This study investigates the critical role of the iron–sulfur (Fe–S) cluster in DOG-1 and its relationship with the cytosolic iron–sulfur protein assembly targeting complex (CTC). We found that a DOG-1 mutant, expected to be defective in Fe–S cluster binding, is primarily localized in the cytoplasm, leading to heightened DNA damage sensitivity and G-rich DNA deletions. We further discovered that the deletion of *mms-19*, a nonessential CTC component, also resulted in DOG-1 sequestered in cytoplasm and increased DNA damage sensitivity. Additionally, we identified that CIAO-1 and CIAO-2B are vital for DOG-1’s stability and repair functions but unlike MMS-19 have essential roles in *C. elegans*. These findings confirm the CTC and Fe–S cluster as key elements in regulating DOG-1, crucial for genome integrity. Additionally, this study advances our understanding of the CTC’s role in Fe–S protein regulation and development in *C. elegans*, offering a model to study its impact on multicellular organism development.

## Graphical Abstract

**Graphical Abstract:**
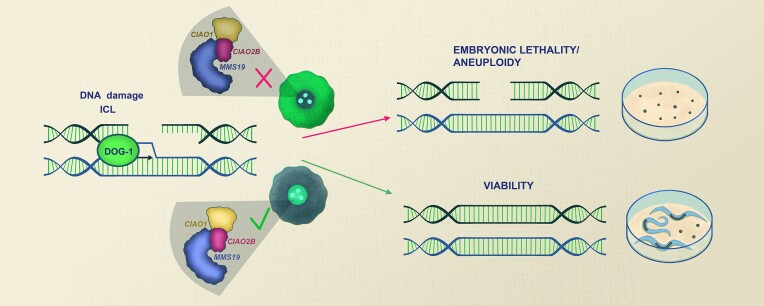

## Introduction

The genome’s integrity is paramount for the proper functioning and preservation of cellular life. The FANCJ/BRIP1 protein plays an instrumental role in protecting genomic fidelity through its involvement in DNA interstrand cross-link (ICL) repair and the resolution of G-quadruplex (G4) structures (1). As a member of the Rad3-like SF2 DNA helicase family, FANCJ/BRIP1 is characterized by a conserved [4Fe–4S] cluster-binding motif comprising four cysteine residues within its helicase domain (2). Pathogenic variants in FANCJ/BRIP1 are associated with Fanconi anemia (FA), a rare hereditary chromosome instability disorder, and are also implicated in hereditary breast and ovarian cancer (3,4). In a seminal study by Odermatt *et al.* (5), it was elucidated that certain FA and cancer-associated mutations in the Fe–S domain adversely affect Fe–S coordination, compromising ICL repair and G4 structure resolution, thereby underscoring the clinical significance of the Fe–S domain (5). FANCJ/BRIP1 is part of a broader ensemble of cytosolic and nuclear Fe–S proteins, several of which are integral to DNA replication and genome maintenance (6,7). The insertion of the Fe–S cluster into FANCJ/BRIP1 is thought to be mediated by the cytosolic iron–sulfur protein assembly (CIA) targeting complex (CTC), which facilitates interaction with apo-proteins in the cytosol (8–10). Studies in human cells suggest that CTC binding to BRIP1/FANCJ is required in iron–sulfur (Fe–S) cluster acquisition and its protein stability (8,10).

FANCJ/BRIP1 was first identified in *Caenorhabditis elegans* as DOG-1 (Deletions Of G-rich DNA), where it plays an essential role in preserving G-rich DNA throughout the genome (11–13). The functional parallels between DOG-1 and FANCJ/BRIP1, particularly in the preservation of G-rich DNA and ICL repair, have been substantiated by subsequent research (11,13). Since the identification of DOG-1 in 2002, genetic studies employing a knockout strain have undeniably established *C. elegans* as an invaluable model for understanding ICL repair, G4 DNA maintenance, and overall molecular mechanisms of FA (11–18).

In this study, we investigate the functional conservation of the Fe–S cluster in DOG-1 by examining a mutant that is expected to be defective in Fe–S cluster binding, as well as mutants in core components of the CTC. We demonstrate a conserved essential role of the Fe–S for proper DOG-1 localization and function. Furthermore, we establish a conserved role of the CTC components, CIAO-1, CIAO-2B and MMS-19, and show that while equally critical for proper DOG-1 localization, stability and function, the CTC components are not equally essential for development in *C. elegans*.

Overall, this study unveils a previously underappreciated facet of DOG-1 regulation predicated on Fe–S acquisition and cellular localization, orchestrated by the CTC. Thus, we have established a valuable model to study the role of Fe–S clusters in other proteins and the role of CTC in a multicellular organism. This study has implications for understanding the molecular intricacies of how Fe–S clusters and the CTC govern cellular processes that are crucial for genomic integrity and the development of a multicellular organism (19,20).

## Materials and methods

### Strains

*Caenorhabditis elegans* strains are obtained from the *Caenorhabditis* Genetics Center (CGC) and/or generated in the lab (Supplementary Table S3). Strains were maintained on *Escherichia coli OP50* seeded NGM plates at 20°C as outlined by Brenner (21), unless reported otherwise. Brood size determination was conducted by placing individual L4 worms on separate plates and transferring them to a new plate every day for 4 days and counting progeny.

### Mutant generation

Clustered regularly interspaced short palindromic repeats (CRISPR) and CRISPR-associated protein 9 (Cas9)-mediated gene editing was used for mutant generation as detailed by Dokshin *et al.* (22). For a list of guide RNAs and single-stranded DNA repair templates, please refer to Supplementary Table S4.

### TMP-UVA assay

One-day-old adults were treated with 10 μg/ml TMP (trioxsalen, Sigma, T6137-100MG) in M9 buffer for 1 h. After TMP treatment, the worms were moved to a fresh plate and exposed to ultraviolet A (UVA; 200–400 J) at an intensity of 300 μW/cm^2^ [J/(s m^2^)]. The treated worms were allowed to recover overnight and then transferred to fresh plates to lay eggs for 4 h (20–24 h after TMP/UVA treatment). The hatch rate was scored 24–48 h after egg laying.

### Western blot analysis

Worm protein lysates were prepared by placing 20 one-day-old adult worms in 20 μl of M9 medium, and then denatured by adding 4× sodium dodecyl sulfate sample buffer and boiling for 10 min. Proteins were separated by sodium dodecyl sulfate–polyacrylamide gel electrophoresis, transferred onto polyvinylidene difluoride membranes, and probed with mouse anti-FLAG (1:10 000, Sigma) and mouse anti-α-tubulin (1:10 000, Sigma). Horseradish peroxidase-conjugated donkey anti-rabbit was used as the secondary antibody. Detection was accomplished using ECL Prime Western Blotting Detection Reagents (Cytiva, RPN2232). Bands were visualized using an Amersham Imager 600 and quantified using ImageJ.

### Cytological preparation and immunostaining

One-day-old adult worms were collected, washed in M9, fixed in cold methanol, rehydrated with phosphate-buffered saline and mounted in SlowFade Gold Antifade Mountant with 4′,6-diamidino-2-phenylindole dihydrochloride (DAPI; Invitrogen). Dissection and immunostaining of gonads were performed using an adapted protocol as described (23). A mouse anti-FLAG (1:500, Sigma) antibody was used to probe FLAG::DOG-1 or its variant expression in the germ cells from gonads dissected from 1-day-old worms. All images were captured on a Zeiss Imager M2 microscope using Zen software. Nucleocytoplasmic distribution of FLAG::DOG-1 was determined using ImageJ line scan analysis.

### Detection of G4 DNA locus deletion

Deletion of endogenous G4 DNA loci was assessed using a polymerase chain reaction (PCR)-based approach as previously described (13,15,24). Protease K-treated worm lysates from single worms were subjected to nested rounds of PCRs with primers that flank a G4 motif. A typical wild-type amplicon is around 1 kb, and smaller PCR bands indicate deletions. The following primers were used to detect a previously characterized G4 locus: *qua830* 5′-CTAGTTCAGGGTATCTGGAC-3′; 5′-GATTGCGGGCACTTTACCTCG-3′; 5′-CCTTCTCTCGAAGCGCGACC-3′; and 5′-GATTTTATTGACTCTCCGTCCG-3′.

## Results

### Fe–S cluster is essential for the DOG-1 function

**Figure 1.**
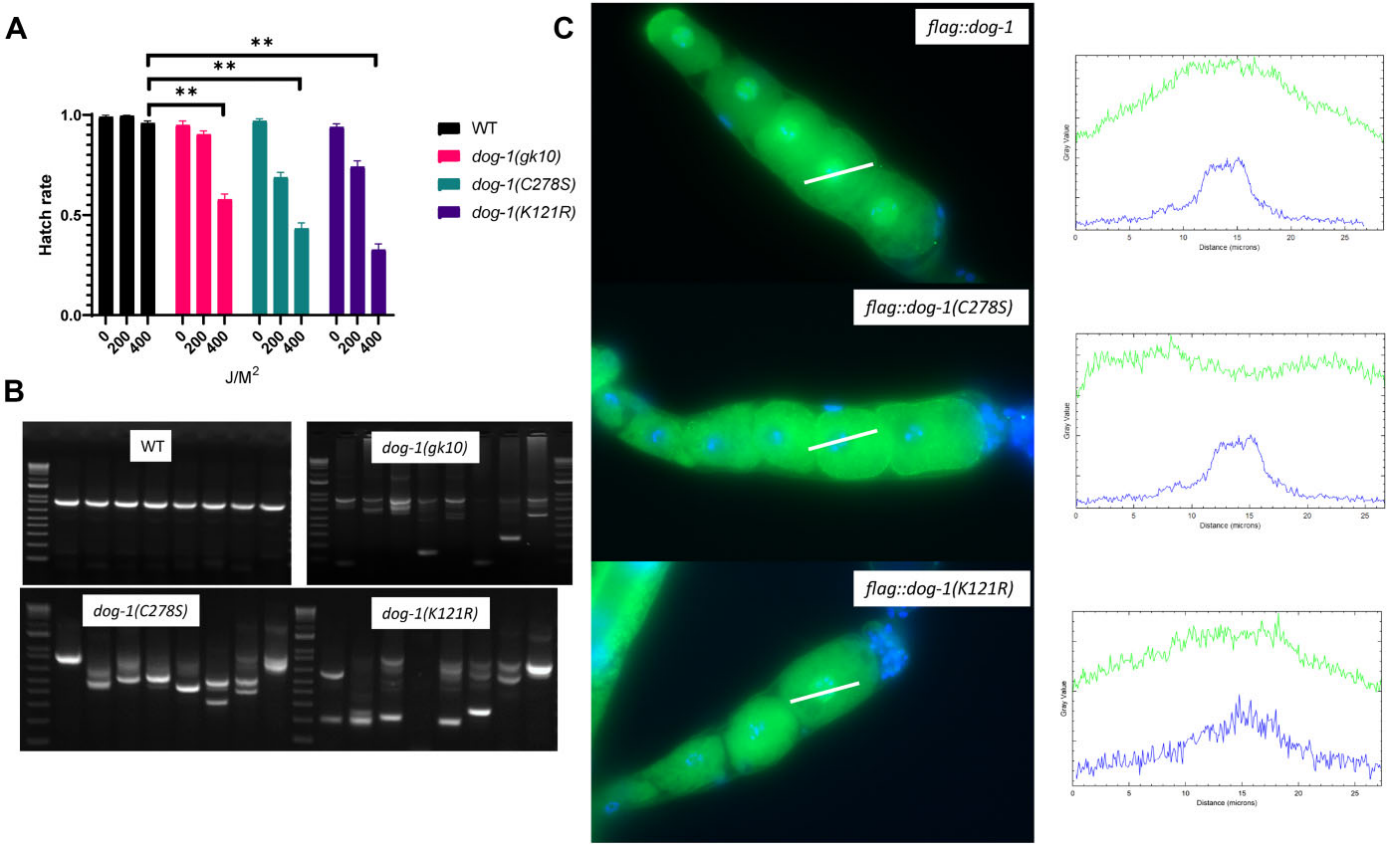
Fe–S cluster is essential for DOG-1 helicase. (**A**) Fe–S binding-defective DOG-1(C278S) is sensitive to TMP/UVA-induced ICLs. Error bars represent the standard error of the mean. (**B**) Guanine-rich DNA at *qua830* locus is frequently deleted in *dog-1(C278S)* worms. (**C**) FLAG::DOG-1(C278S) is not imported in the nucleus and is sequestered in the cytosol of *C. elegans* oocytes. Line scan analysis across an oocyte reveals the nucleocytoplasmic distribution of FLAG::DOG-1 (upper) relative to the nucleus (bottom).

Like human BRIP1/FANCJ, DOG-1 contains a conserved helicase domain and an Fe–S binding domain (Supplementary Figure S1A). To directly understand the significance of Fe–S in DOG-1, we generated a *dog-1(C278S)* mutant through CRISPR/Cas9-mediated homologous recombination repair. In this mutant, a conserved cysteine (C278) coordinating Fe–S is changed to a serine (Supplementary Figure S1A). This alteration is likely to render DOG-1(C278S) incapable of binding to the Fe–S cluster based on previous studies (5). Concurrently, we created a helicase-deficient *dog-1* mutant, *dog-1(K121R)*, to serve as a control (Supplementary Figure S1A). We assessed the proficiency of these mutants in repairing ICLs, employing an established assay in which a deletion mutant *dog-1(gk10)* has previously exhibited a heightened sensitivity to TMP/UVA (11). We discovered that both *dog-1(C278S)* and *dog-1(K121R)* are sensitive to UVA/TMP treatment at a level comparable to the *dog-1(gk10)* knockout, indicating that Fe–S is likely essential for DOG-1 function in ICL repair (Figure 1A). We also explored the impact of Fe–S on the integrity of guanine-rich DNA regions. We measured the deletion frequency of a previously well-characterized guanine-rich DNA locus *qua830*, which forms a G4 structure and is frequently deleted in *dog-1(gk10)* worms (Figures 1B and 4F, and Supplementary Table S1) (15,25). Notably, both *dog-1(C278S)* and *dog-1(K121R)* mutants presented with frequent deletions at the *qua830* locus, showing the necessity of Fe–S in maintaining guanine-rich DNA sequences (Figure 1B and Supplementary Table S1). To track the cellular localization of DOG-1, we inserted a sequence that encodes a FLAG tag into the N-terminus of the endogenous *dog-1* gene (*3xflag::dog-1*) (Supplementary Figure S1A). First, we confirmed that the insertion of the *3xflag* sequence does not change the normal function of DOG-1 as *3xflag::dog-1* worms repair ICLs at wild-type levels (Supplementary Figure S1D). Next, we showed that FLAG::DOG-1 predominantly localizes in the nucleus and associates with DNA, in line with its role as DNA helicase (Supplementary Figure S1C). We then introduced both *C278S* and *K121R* mutations in the *3xflag::dog-1* strain through CRISPR/Cas9-mediated homologous recombination repair. We found that FLAG::DOG-1(C278S) is reduced in total worm lysates, while FLAG::DOG-1(K121R) level is not changed (Supplementary Figure S1B). We carried out immunostaining and imaging of the dissected gonads using the same conditions across the genotypes and found that comparable levels of FLAG::DOG-1(C278S) were present in germline cells, but localized in the cytoplasm (Figure 1C). In contrast, both wild-type DOG-1 and helicase-deficient DOG-1(K121R) displayed normal nuclear localization (Figure 1C). This striking observation suggests that the expected Fe–S binding deficiency results in DOG-1 being sequestered in the cytoplasm, preventing its importation into the nucleus. Furthermore, the Fe–S cluster may play a role in maintaining the protein stability of DOG-1. In conclusion, our results underscore a conserved and essential role for the C278 in the functioning of DOG-1, especially in ICL repair and maintaining the integrity of guanine-rich DNA regions.

### Differential requirement of CTC components for viability in *C. elegans*

**Figure 2.**
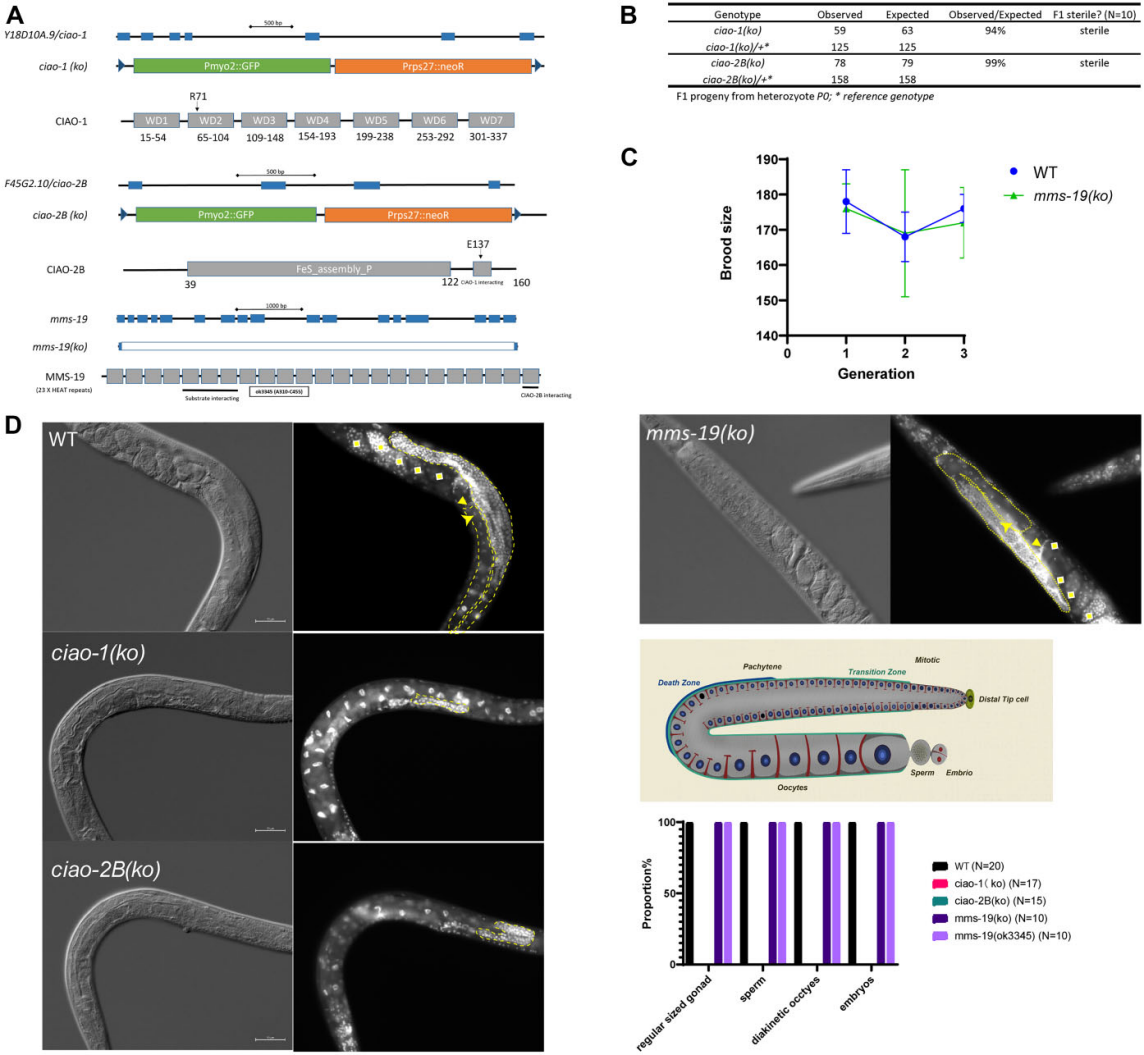
Differential requirement of CTC genes for viability in *C. elegans*. (**A**) Schematic representation of *ciao-1, ciao-2B* and *mms-19* genes and mutations. (**B**) *ciao-1(ko)* and *ciao-2B(ko)* homozygotes are viable but sterile. F1 progeny is produced by P0 heterozygotes. (**C**) Normal brood size for *mms-19(ko)* over three generations. (**D**) Reduced germline population (outlined by dotted lines), lack of mature oocytes (arrows), sperms (triangles) and embryos (diamonds) in *ciao-1* and *ciao-2B* and normal germlines in *mms-19(ko)* worms.

Building on the understanding that the Fe–S cluster is vital for DOG-1’s function, we next wanted to explore how DOG-1 might interact with the CTC. We hypothesized that DOG-1, which is expected to be unable to bind Fe–S (as in the DOG-1(C278S) variant), could still associate with the CTC but might not be released as an active protein. To assess the involvement of the CTC in modulating DOG-1 activity, it was crucial to investigate the unexplored function of CTC components in *C. elegans*. Based on protein sequence homology, we were able to identify Y18D10A.9, F45G2.10 and MMS-19 as orthologues of CIAO1, CIAO2B and MMS19, respectively. For clarity in further discussions, *Y18D10A.9* was renamed to *ciao-1* and *F45G2.10* to *ciao-2B* (Figure 2A and Supplementary Table S2). We obtained and generated CRISPR knockout alleles for these genes: *ciao-1(gk5013)*, *ciao-2B(gk5482)* and *mms-19(ko)*, subsequently referred to as *ciao-1(ko)*, *ciao-2B(ko)* and *mms-19(ko)* (Figure 2A and Supplementary Table S2).

#### ciao-1 and ciao-2B are required for viability and fertility, while mms-19 is dispensable

The F1 homozygotes of *ciao-1(ko)* or *ciao-2B(ko)* developed into adults, likely due to maternal contributions, but exhibited 100% sterility and failed to produce embryos. As a result, these mutants display maternal effect sterility (Figure 2B). This suggests that *ciao-1* and *ciao-2B* are essential for *C. elegans*’ development. In contrast, *mms-19(ko)* homozygotes were viable, exhibiting normal brood size and hatch rate under standard culture conditions (Figure 2C and Supplementary Table S2).

#### ciao-1 and ciao-2B mutants exhibit impaired germline development

We further analyzed the gonads of *ciao-1(ko)* and *ciao-2B(ko)* worms and found them to be smaller than normal. Staining with DAPI revealed a diminished population of germ cells in young adult worms. Moreover, mature sperms, oocytes and embryos were absent in the gonads of these mutants (Figure 2D). In contrast, *mms-19(ko)* worms showed normal-sized gonads and successful development of sperms, oocytes and embryos (Figure 2D). These findings suggest different requirements of CTC components in fertility and propagation.

### MMS-19 is required for ICL repair by regulating DOG-1 stability and nuclear localization

#### mms-19 is required for ICL repair

**Figure 3.**
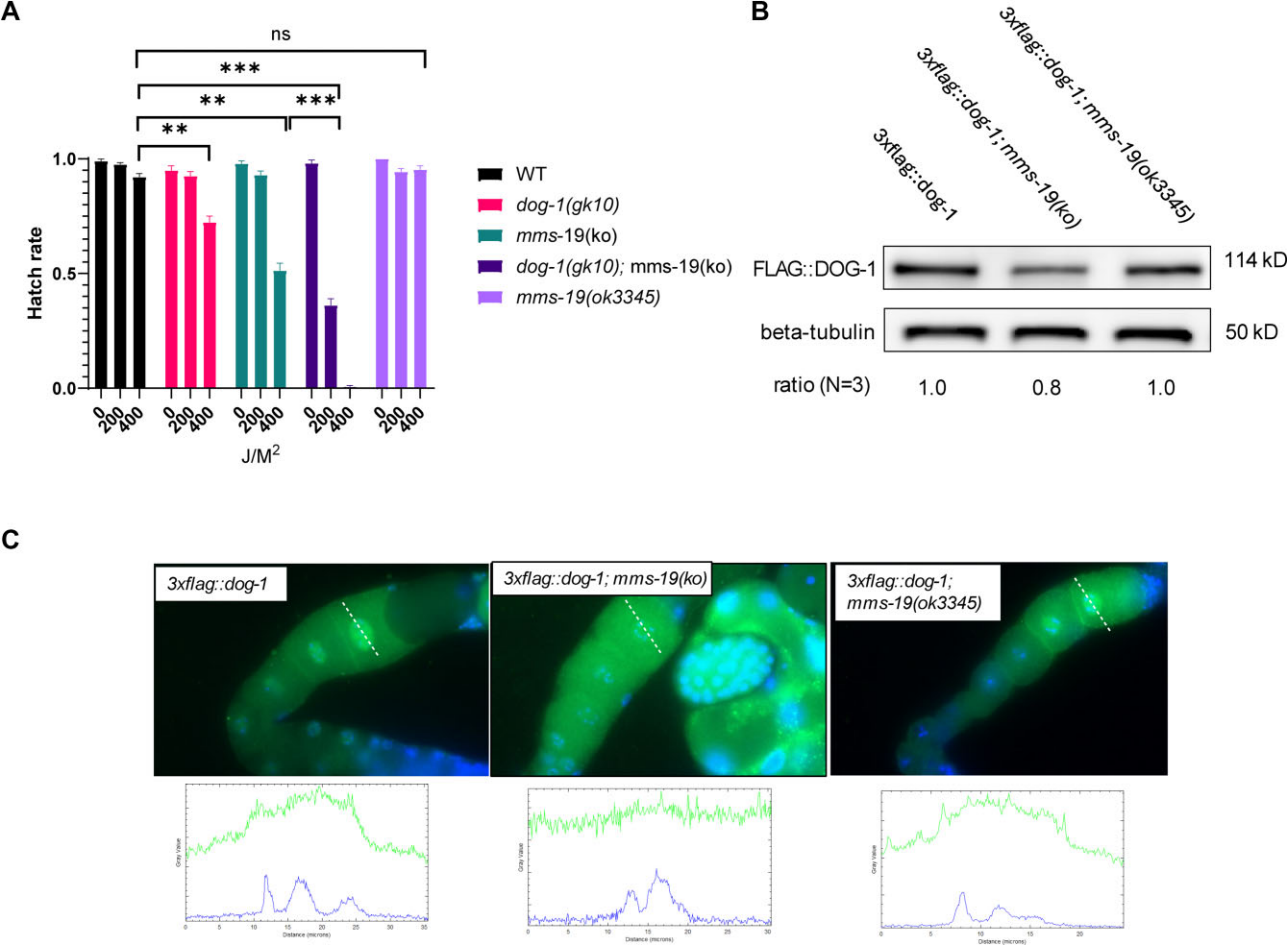
MMS-19 is required for ICL repair by regulating DOG-1 stability and nuclear localization. (**A**) *mms-19* is required for ICL repair. (**B**) FLAG::DOG-1 is reduced in *mms-19(ko)* total worm lysate. (**C**) Diminished nuclear FLAG::DOG-1 in germline cells and embryos of *mms-19(ko)* worms. Line scan analysis across an oocyte reveals the nucleocytoplasmic distribution of FLAG::DOG-1 (upper) relative to the nucleus (bottom).

As *ciao-1(ko)* and *ciao-2B(ko)* homozygotes could not be propagated, our initial investigation on DOG-1 function in these mutant worms focused on the viable *mms-19(ko)* mutants. Our findings revealed that *mms-19(ko)* worms display a sensitivity level to TMP/UVA comparable to that of *dog-1(gk10)* worms, with *dog-1(gk10);mms-19(ko)* double mutants demonstrating an even greater sensitivity to TMP/UVA treatment (Figure 3A). We attribute this enhanced sensitivity of the double mutant to an additive effect of partial loss of function of DOG-1 and compromised function of other Fe–S proteins that may require MMS-19.

#### MMS-19 is required for proper nuclear localization of DOG-1

In the absence of MMS-19, FLAG::DOG-1 is slightly reduced in the total worm lysates (Figure 3B). Interestingly, FLAG::DOG-1 appeared to be evenly distributed between the cytosol and nucleus in *mms-19(ko)* oocytes, suggesting either cytosolic retention or impaired nuclear import (Figure 3C). A similar reduction in nuclear localization was observed in *mms-19(ko)* embryos (Supplementary Figure S3A). This cellular phenotype resembles, to an extent, the distribution of DOG-1(C278S) mutant, supporting the lack of ability of the DOG-1(C278S) to bind Fe–S (Figure 1C). This similarity suggests that MMS-19 might facilitate the release of mature DOG-1 and/or assist in its nuclear import/export. Without MMS-19, DOG-1 seems to be confined to the cytosol, possibly undergoing proteasomal degradation, as suggested by the slightly reduced FLAG::DOG-1 levels and diminished immunostaining in the embryos (Figure 3B and Supplementary Figure S3A). However, this phenotype was not as pronounced as that observed for the DOG-1(C278S) mutation (Figures 1C and 3C, and Supplementary Figure S3A). The partial nuclear localization of DOG-1 may indicate that some mature DOG-1 proteins are made and transported into the nucleus, explaining a lack of G-DNA deletions in the *mms-19(ko)* worms (Supplementary Figure S3B and Supplementary Table S1).

We further examined another *mms-19* deletion mutant, an *mms-19(ok3345)* generated previously by others and available from the CGC but otherwise uncharacterized. We found that this mutant resulted in an in-frame deletion of amino acids (310–455) in MMS-19 (Supplementary Figure S3C and D). Despite this deletion and a temperature-dependent ‘mortal germline (Mrt)’ phenotype (26) (Supplementary Figure S2A), the substrate-binding domain and the CIAO-2B binding domain remained unaffected (Figure 2A), indicating that it could still interact with DOG-1 and function as part of the CTC. Consistent with this, we found normal sensitivity to TMP/UVA treatment and normal nuclear localization (Figure 3A and B, and Supplementary Figure S3B), supporting the idea that the substrate-binding domain and CIAO-2B binding domain in MMS-19 are sufficient for DOG-1 maturation and release in this mutant.

### CIAO-1 and CIAO-2B are also required for DOG-1 stability, ICL repair and G-rich sequence maintenance

#### ciao-1 and ciao-2B are required for DOG-1 stability

**Figure 4.**
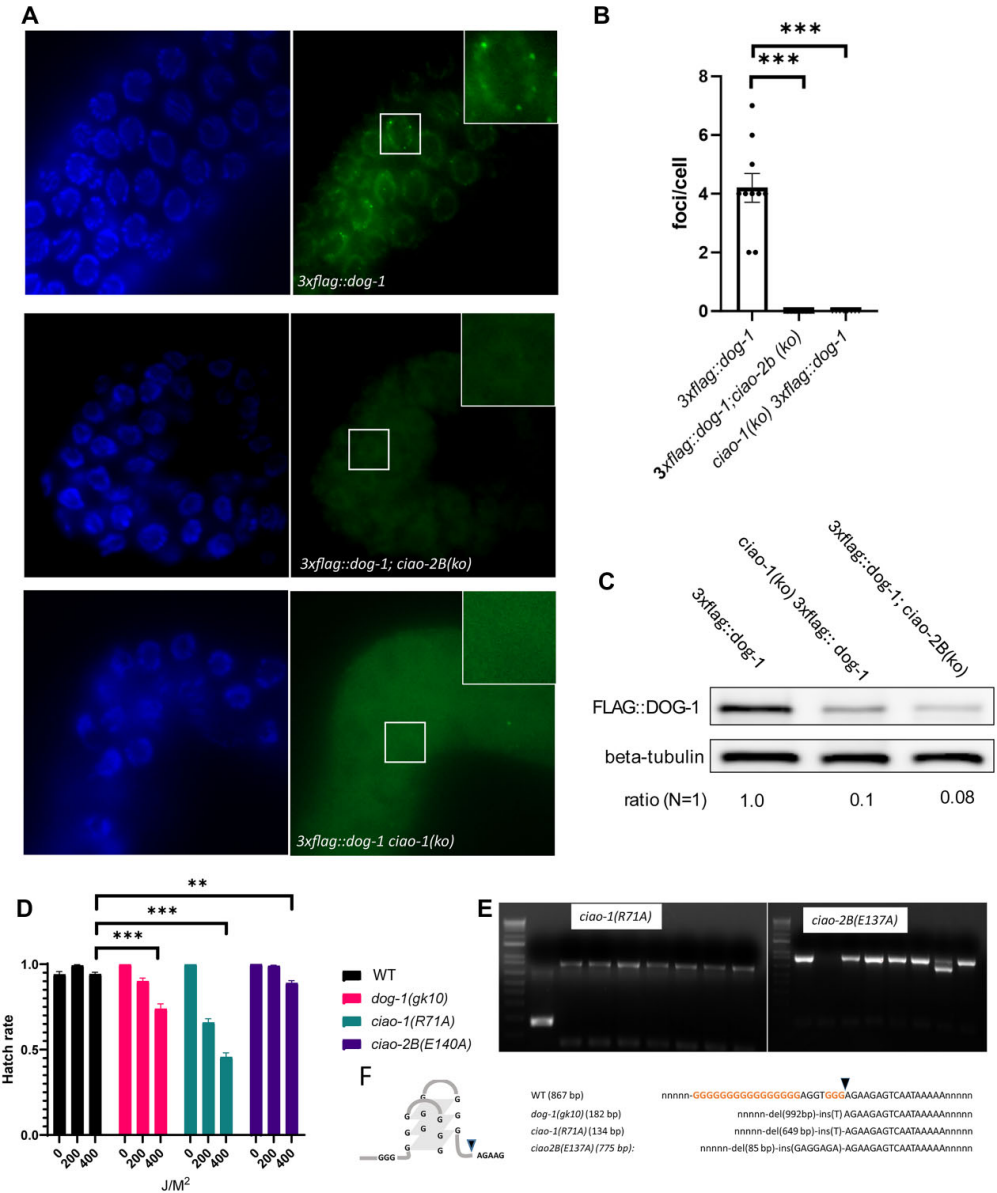
CIAO-1 and CIAO-2B are also required for DOG-1 stability, ICL repair and G-rich sequence maintenance. (**A**) Lack of DOG-1 foci in *ciao-1* and *ciao-2B* germline cells. (**B**) Quantification of DNA-associated foci. Error bars represent the standard error of the mean. (**C**) Immunoblot of FLAG::DOG-1 *ciao-1* and *ciao-2B* worm lysates. (**D**) *ciao-1(R71A)* and *ciao-2B(E137A)* mutants sensitive to TMP/UVA treatment. (**E**) G-rich DNA deletion is observed in *ciao-1(R71A)* and *ciao-2B(E137A)* mutants. (**F**) Sanger sequencing confirms the G-tract deletion found in panel (E), resembling characteristic deletions found in *dog-1(gk10)* worm.

Encouraged by our findings with *mms-19*, we sought to understand the potential roles of CIAO-1 and CIAO-2B in regulating DOG-1 stability and cellular localization. Through meticulous collection, we obtained a sufficient number of the otherwise infertile *ciao-1(ko)* and *ciao-2B(ko)* F1 worms. Analysis of these mutants showed a stark reduction in FLAG::DOG-1 levels in germ cells, with an absence of the DNA-associated foci normally seen in wild-type germ cells (Figure 4A and B) and significant depletion of FLAG::DOG-1 in total worm lysates (Figure 4C). These data imply that when CIAO-1 or CIAO-2B binds to DOG-1, it may protect DOG-1 from breaking down. This is suggested by the still noticeable levels of a form of DOG-1, FLAG::DOG-1(C278S), in germ cells (Figure 1C). These results are consistent with earlier findings that some Fe–S cluster proteins are less stable without CTC genes (10,27).

#### ciao-1 and ciao-2B play a role in ICL repair and guanine-rich DNA maintenance

The sterility of *ciao-1(ko)* and *ciao-2B(ko)* homozygotes prevented us from testing their sensitivity to UVA/TMP treatment. We generated missense variants *ciao-1(R71A)* and *ciao-2B(E137A)* to circumvent this. Based on the recent crystal structure, R71 and E137 are positioned at the interaction interface of CIAO-1 and CIAO-2B and are predicted to interrupt CIAO-1 and CIAO-2B interaction (Supplementary Figure S4A) (28). The homozygous mutants of *ciao-1(R71A)* and *ciao-2B(E137A)* showed no apparent phenotypes and are viable (Supplementary Table S2). However, we noticed that *ciao-2B(E137A)* had a lower hatching rate at 25°C, indicating that it is sensitive to higher temperature (Supplementary Figure S2B and Supplementary Table S2). The DOG-1 protein levels and cellular location seemed normal in *ciao-1(R71A)* and *ciao-2B(E137A)* worms (Supplementary Figure S4B and C). We then tested the sensitivity to TMP/UVA treatment at 23°C, a permissive temperature at which untreated *ciao-2B(E137A)* worms hatch normally. We found *ciao-1(R71A)* worms to be sensitive to TMP/UVA treatment at levels comparable to *dog-1(gk10)*, and *ciao-2B(E137A)* also showed sensitivity, albeit to a lesser extent (Figure 4D). These results were further supported when sensitivity to TMP/UVA treatment was tested in strains carrying the double mutations *dog-1(gk10) ciao-1(R71A)* or *dog-1(gk10);ciao-2B(E137A)*, as shown in Supplementary Figure S5A.

We examined whether guanine-rich DNA is unstable in *ciao-1(R71A)* and *ciao-2B(E137A)* worms. We observed deletions of guanine-rich DNA in both *ciao-1(R71A)* and *ciao-2B(E137A)* worms (Figure 4E and Supplementary Table S1). Although the frequency of deletions is rare and not statistically significant when compared to the wild-type strain, we confirmed a unique and highly specific deletion signature by Sanger sequencing (13,24) (Figure 4F). In addition, we did not observe a notable increase in the G4 tract deletion frequency in the strains corresponding to the double mutants *dog-1(gk10) ciao-1(R71A)* and *dog-1(gk10);ciao-2B(E137A)* compared to the deletion frequency detected for the *dog-1(gk10)* knockout strain (Supplementary Figure S5B and Supplementary Table S1). Thus, we infer that these two mutations have minimal effects on G4 tract stability.

Overall, we conclude that, like MMS-19, the proper function of CIAO-1 and CIAO-2B is also required to maintain the appropriate DOG-1 protein level and carry out ICL repair to maintain genome stability.

## Discussion

### Conserved requirement of Fe–S for DOG-1

Our research demonstrated that Fe–S is indispensable for the DNA helicase DOG-1, particularly concerning its crucial functions in repairing ICLs and in maintaining G-rich DNA sequences. The *dog-1* mutant, which is presumably defective in Fe–S binding, mimics the *dog-1* deletion mutant and helicase-inactive mutant in experiments involving UVA/TMP and G-tract deletions.

### Cellular localization of DOG-1 in response to defective Fe–S binding or insertion by CTC

Our study uncovered a novel finding that DOG-1 is mislocalized when there is a defect in the CTC itself, as in the case of *mms-19(ko)*, or the DOG-1 itself cannot accept Fe–S cluster, as expected in the case of DOG-1(C278S). We hypothesize that the CTC might initially bind DOG-1 tightly prior to inserting Fe–S, leading to confinement of DOG-1 in the cytosol. MMS-19 could play a role in the effective release of mature DOG-1 from the CTC and/or its import/export balance in and out of the nucleus. Without MMS-19, DOG-1 may still acquire Fe–S and enter the nucleus to some extent, but it may not be efficiently released from the CTC and/or imported into the nucleus. Thus, without MMS-19, some DOG-1 must still be able to receive Fe–S and get into the nucleus: this is supported by FLAG::DOG-1 seen in the cell nucleus of *mms-19(ko)* and no G-rich DNA deletions being detected in *mms-19(ko)* worms. However, DOG-1 may not be efficiently released from the CTC and/or its import/export in and out of the nucleus may be affected in the absence of MMS-19.

### DOG-1 protein stability in response to defective Fe–S binding or insertion by CTC

Previous studies found that human and yeast MMS-19 interact with BRIP1/FANCJ and Rad3, respectively, and BRIP1’s protein levels decrease when MMS-19 is depleted. Our study shows that CIAO-1, CIAO-2B and, to a lesser extent, MMS-19 are necessary for the stability of DOG-1 DNA helicase. Furthermore, we noticed lower total protein levels of the presumably Fe–S binding-defective DOG-1(C278S), which supports the hypothesis that proper Fe–S integration is crucial for DOG-1 stability.

### Repair of TMP/UVA-induced ICLs

We observed sensitivities to TMP/UVA treatment for *mms-19(ko)*, *ciao-1(R71A)* and *ciao-2B(E137A)* as well as in the predicted Fe–S binding-defective mutant *dog-1(C278S)*. Thus, we attribute the increased sensitivity at least partially to the loss of DOG-1 function due to CTC deficiencies. However, it is essential to recognize that the CTC is known to be involved in integrating Fe–S into a variety of DNA repair proteins within the cytoplasm and nucleus (6). Therefore, mutations in *ciao-1*, *ciao-2B* or *mms-19* could set off a chain reaction of effects that might either directly or indirectly influence the assessments of DOG-1 functions. For example, the double mutant *dog-1(gk10);mms-19(ko)* exhibits substantially higher sensitivity to TMP/UVA treatment compared to either *dog-1(gk10)* or *mms-19(ko)* single mutants. This could be explained by the additive effect with partial loss of DOG-1 function and the loss of function of another Fe–S DNA repair protein such as *rtel-1* (29). However, although the double mutant *dog-1(gk10) ciao-1(R71A)* also exhibited higher sensitivity to the TMP/UVA treatment than the *dog-1(gk10)* single mutant, the effect was less robust than that observed for the double mutant *dog-1(gk10);mms-19(ko)*, whereas the *dog-1(gk10);ciao-2B(E137A)* double mutant sensitivity to TMP/UVA-induced ICLs was comparable to that of the *dog-1(gk10)* single mutant. These results are consistent with the phenotypic differences observed for the strains containing *mms-19(ko)* versus *ciao-1(R71A)* or *ciao-2B(E137A)* single mutations regarding DOG-1 protein levels and cellular localization.

### Maintenance of G-rich DNA

The FANCJ/BRIP1 DNA helicase promotes DNA synthesis through G4 structures, which are stable secondary structures formed by G-rich DNA sequences (30,31). In *C. elegans*, loss of DOG-1 results in deletions of G4 forming homopolymeric dC/dGs (G4 DNA-induced deletions) (13,24). G4 DNA-induced deletion is highly specific to DOG-1 loss of function, as a large genome-wide mutagenesis genetic screen aiming to identify additional mutations leading to G-tract deletion only identified additional *dog-1* alleles and no other genes with similar function (18). In our study, we did not observe any deletion events in the viable *mms-19(ko)* mutants. This result suggests that residual mature DOG-1 is still present in the nucleus as discussed earlier. Despite observing the loss of DOG-1 protein in *ciao-1(ko)* and *ciao-2B(ko)* worms (Figure 4A and C), our efforts using single worm PCR (Supplementary Table S1) and bulk analysis (not shown) failed to detect G-tract deletions. This outcome might stem from restricted DNA replication and proliferation in these worms (Figure 2D), possibly hindering the initial formation of deletions since G-tract deletion generation is reliant on DNA replication and mitosis (25). Alternatively, it could be attributed to the possibility of larger deletions occurring beyond the detection capacity of the nested PCR method applied in these worms. We also failed to detect G-tract deletion events in double *ciao-1(R71A);ciao-2B(E137A)* mutants (Supplementary Table S1). This could potentially be explained by the fact that *ciao-1(R71A)* is capable of rescuing the temperature-dependent lethality associated with *ciao-2B(E137A)* (Supplementary Figure S2B). Consequently, the double mutant seems to behave similarly to the wild type rather than exhibiting an exacerbated phenotype. This observation, combined with the finding that the in-frame deletion of *mms-19(ok3345*) did not lead to TMP/UV-induced ICL sensitivity (Figure 3A) nor G-tract deletions (Supplementary Table S1) but did result in an Mrt phenotype (Supplementary Figure S2), underscores the intricate and complex interplay between the various components of the CIA machinery and their diverse substrates.

### Essentiality of CTC genes

CIAO-1 is an essential gene in yeast, flies and plants (32–34). Our results in worms show that *ciao-1(ko)* homozygous progeny produced from heterozygous mothers can survive to sterile adults with a reduced number of germ cells and a lack of embryos. RNA interference depletion of both maternal and zygotic transcripts leads to 100% embryonic lethality (35). Taken together, we conclude that *ciao-1* is also an essential gene in worms. No CIAO-2B mutants in other model organisms have been reported previously. Our results indicate that similar to *ciao-1*, *ciao-2B* is an essential gene required for germline and embryonic development in *C. elegans*. Though *mms-19* knockout mice die at the pre-implantation stage, *mms-19* is not an essential gene in yeast and plants (33,36,37) Worm *mms-19(ko)* is phenotypically normal at standard culture conditions over generations, and thus unlike *ciao-1* and *ciao-2B*, *mms-19* is not required for viability. The CTC interacts with numerous cytosolic and nuclear Fe–S client proteins (6). We postulated that a differential requirement of each of the CTC components for maturing of these client proteins underlines the difference in the essentiality of *mms-19*. Specifically, MMS-19 may play a regulatory role in the release and transport of mature Fe–S proteins.

In conclusion, our study provides a novel understanding of the intricate dynamics among the CTC, Fe–S binding and the DOG-1 DNA helicase, elucidating their roles in DNA repair, protein stability and cellular localization. This multicellular organism model not only unravels the functional nuances of the CTC but also offers a valuable platform to dissect its regulatory mechanisms, significantly contributing to the broader comprehension of DNA repair and cellular homeostasis. In particular, the recently reported implication of CTC genes *ciao-1* and *mms-19* in the onset of lethal neurodegenerative diseases (19,20) highlights the significance of the work presented herein in assisting the study of human disease mechanisms.

## Supplementary Material

gkae617_Supplemental_File

## Data Availability

The data underlying this article are available in the article and in its online supplementary material.

## References

[1] Bharti S.K. , Sommers J.A., George F., Kuper J., Hamon F., Shin-ya K., Teulade-Fichou M.P., Kisker C., Brosh R.M. Jr Specialization among iron–sulfur cluster helicases to resolve G-quadruplex DNA structures that threaten genomic stability. J. Biol. Chem. 2013; 288:28217–28229. 23935105 10.1074/jbc.M113.496463 PMC3784731

[2] Rudolf J. , Makrantoni V., Ingledew W.J., Stark M.J., White M.F. The DNA repair helicases XPD and FancJ have essential iron–sulfur domains. Mol. Cell. 2006; 23:801–808. 16973432 10.1016/j.molcel.2006.07.019

[3] Calvo J.A. , Fritchman B., Hernandez D., Persky N.S., Johannessen C.M., Piccioni F., Kelch B.A., Cantor S.B. Comprehensive mutational analysis of the BRCA1-associated DNA helicase and tumor-suppressor FANCJ/BACH1/BRIP1. Mol. Cancer Res. 2021; 19:1015–1025. 33619228 10.1158/1541-7786.MCR-20-0828 PMC8178215

[4] Suhasini A.N. , Brosh R.M. Jr Disease-causing missense mutations in human DNA helicase disorders. Mutat. Res. 2013; 752:138–152. 23276657 10.1016/j.mrrev.2012.12.004 PMC3640642

[5] Odermatt D.C. , Lee W.T.C., Wild S., Jozwiakowski S.K., Rothenberg E., Gari K. Cancer-associated mutations in the iron–sulfur domain of FANCJ affect G-quadruplex metabolism. PLoS Genet. 2020; 16:e1008740. 32542039 10.1371/journal.pgen.1008740 PMC7316351

[6] Braymer J.J. , Freibert S.A., Rakwalska-Bange M., Lill R. Mechanistic concepts of iron–sulfur protein biogenesis in biology. Biochim. Biophys. Acta Mol. Cell Res. 2021; 1868:118863. 33007329 10.1016/j.bbamcr.2020.118863

[7] Wachnowsky C. , Fidai I., Cowan J.A. Iron–sulfur cluster biosynthesis and trafficking—impact on human disease conditions. Metallomics. 2018; 10:9–29. 29019354 10.1039/c7mt00180k PMC5783746

[8] Gari K. , Leon Ortiz A.M., Borel V., Flynn H., Skehel J.M., Boulton S.J MMS19 links cytoplasmic iron–sulfur cluster assembly to DNA metabolism. Science. 2012; 337:243–245. 22678361 10.1126/science.1219664

[9] Paul V.D. , Lill R. Biogenesis of cytosolic and nuclear iron–sulfur proteins and their role in genome stability. Biochim. Biophys. Acta. 2015; 1853:1528–1539. 25583461 10.1016/j.bbamcr.2014.12.018

[10] Stehling O. , Vashisht A.A., Mascarenhas J., Jonsson Z.O., Sharma T., Netz D.J., Pierik A.J., Wohlschlegel J.A., Lill R. MMS19 assembles iron–sulfur proteins required for DNA metabolism and genomic integrity. Science. 2012; 337:195–199. 22678362 10.1126/science.1219723 PMC3420340

[11] Youds J.L. , Barber L.J., Ward J.D., Collis S.J., O’Neil N.J., Boulton S.J., Rose A.M. DOG-1 is the *Caenorhabditis elegans* BRIP1/FANCJ homologue and functions in interstrand cross-link repair. Mol. Cell. Biol. 2008; 28:1470–1479. 18086896 10.1128/MCB.01641-07 PMC2258786

[12] Wu Y. , Shin-ya K., Brosh R.M. Jr FANCJ helicase defective in Fanconi anemia and breast cancer unwinds G-quadruplex DNA to defend genomic stability. Mol. Cell. Biol. 2008; 28:4116–4128. 18426915 10.1128/MCB.02210-07 PMC2423121

[13] Cheung I. , Schertzer M., Rose A., Lansdorp P.M. Disruption of dog-1 in *Caenorhabditis elegans* triggers deletions upstream of guanine-rich DNA. Nat. Genet. 2002; 31:405–409. 12101400 10.1038/ng928

[14] Wilson D.M. , Rieckher M., Williams A.B., Schumacher B. Systematic analysis of DNA crosslink repair pathways during development and aging in *Caenorhabditis elegans*. Nucleic Acids Res. 2017; 45:9467–9480. 28934497 10.1093/nar/gkx660 PMC5766164

[15] Koole W. , van Schendel R., Karambelas A.E., van Heteren J.T., Okihara K.L., Tijsterman M. A polymerase theta-dependent repair pathway suppresses extensive genomic instability at endogenous G4 DNA sites. Nat. Commun. 2014; 5:3216. 24496117 10.1038/ncomms4216

[16] Jones M. , Rose A. A DOG’s view of Fanconi anemia: insights from *C. elegans*. Anemia. 2012; 2012:323721. 22690333 10.1155/2012/323721 PMC3368526

[17] Maizels N. Genomic stability: FANCJ-dependent G4 DNA repair. Curr. Biol. 2008; 18:R613–R614. 18644339 10.1016/j.cub.2008.06.011 PMC3806494

[18] Kruisselbrink E. , Guryev V., Brouwer K., Pontier D.B., Cuppen E., Tijsterman M. Mutagenic capacity of endogenous G4 DNA underlies genome instability in FANCJ-defective *C. elegans*. Curr. Biol. 2008; 18:900–905. 18538569 10.1016/j.cub.2008.05.013

[19] Maio N. , Orbach R., Zaharieva I., Topf A., Donkervoort S., Munot P., Mueller J., Willis T., Verma S., Peric S. et al. Loss of function of the cytoplasmic Fe–S assembly protein CIAO1 causes a neuromuscular disorder with compromise of nucleocytoplasmic Fe–S enzymes. 2023; medRxiv doi:20 December 2023, preprint: not peer reviewed 10.1101/2023.12.20.23300170. PMC11178529 38950322

[20] van Karnebeek C.D.M. , Tarailo-Graovac M., Leen R., Meinsma R., Correard S., Jansen-Meijer J., Prykhozhij S.V., Pena I.A., Ban K., Schock S. et al. CIAO1 and MMS19 deficiency: a lethal neurodegenerative phenotype caused by cytosolic Fe–S cluster protein assembly disorders. Genet. Med. 2024; 26:101104. 38411040 10.1016/j.gim.2024.101104 PMC11788579

[21] Brenner S. The genetics of *Caenorhabditis elegans*. Genetics. 1974; 77:71–94. 4366476 10.1093/genetics/77.1.71 PMC1213120

[22] Dokshin G.A. , Ghanta K.S., Piscopo K.M., Mello C.C. Robust genome editing with short single-stranded and long, partially single-stranded DNA donors in *Caenorhabditis elegans*. Genetics. 2018; 210:781–787. 30213854 10.1534/genetics.118.301532 PMC6218216

[23] Phillips C.M. , McDonald K.L., Dernburg A.F. Cytological analysis of meiosis in *Caenorhabditis elegans*. Methods Mol. Biol. 2009; 558:171–195. 19685325 10.1007/978-1-60761-103-5_11 PMC3644504

[24] Youds J.L. , O’Neil N.J., Rose A.M. Homologous recombination is required for genome stability in the absence of DOG-1 in *Caenorhabditis elegans*. Genetics. 2006; 173:697–708. 16547095 10.1534/genetics.106.056879 PMC1526509

[25] Lemmens B. , van Schendel R., Tijsterman M. Mutagenic consequences of a single G-quadruplex demonstrate mitotic inheritance of DNA replication fork barriers. Nat. Commun. 2015; 6:8909. 26563448 10.1038/ncomms9909 PMC4654259

[26] Ahmed S. , Hodgkin J. MRT-2 checkpoint protein is required for germline immortality and telomere replication in *C. elegans*. Nature. 2000; 403:159–164. 10646593 10.1038/35003120

[27] van Wietmarschen N. , Moradian A., Morin G.B., Lansdorp P.M., Uringa E.J. The mammalian proteins MMS19, MIP18, and ANT2 are involved in cytoplasmic iron–sulfur cluster protein assembly. J. Biol. Chem. 2012; 287:43351–43358. 23150669 10.1074/jbc.M112.431270 PMC3527922

[28] Kassube S.A. , Thoma N.H. Structural insights into Fe–S protein biogenesis by the CIA targeting complex. Nat. Struct. Mol. Biol. 2020; 27:735–742. 32632277 10.1038/s41594-020-0454-0

[29] Barber L.J. , Youds J.L., Ward J.D., McIlwraith M.J., O’Neil N.J., Petalcorin M.I., Martin J.S., Collis S.J., Cantor S.B., Auclair M. et al. RTEL1 maintains genomic stability by suppressing homologous recombination. Cell. 2008; 135:261–271. 18957201 10.1016/j.cell.2008.08.016 PMC3726190

[30] Bosch P.C. , Segura-Bayona S., Koole W., van Heteren J.T., Dewar J.M., Tijsterman M., Knipscheer P FANCJ promotes DNA synthesis through G-quadruplex structures. EMBO J. 2014; 33:2521–2533. 25193968 10.15252/embj.201488663 PMC4282361

[31] Wu C.G. , Spies M. G-quadruplex recognition and remodeling by the FANCJ helicase. Nucleic Acids Res. 2016; 44:8742–8753. 27342280 10.1093/nar/gkw574 PMC5062972

[32] Balk J. , Aguilar Netz D.J., Tepper K., Pierik A.J., Lill R. The essential WD40 protein Cia1 is involved in a late step of cytosolic and nuclear iron–sulfur protein assembly. Mol. Cell. Biol. 2005; 25:10833–10841. 16314508 10.1128/MCB.25.24.10833-10841.2005 PMC1316972

[33] Luo D. , Bernard D.G., Balk J., Hai H., Cui X. The DUF59 family gene AE7 acts in the cytosolic iron–sulfur cluster assembly pathway to maintain nuclear genome integrity in *Arabidopsis*. Plant Cell. 2012; 24:4135–4148. 23104832 10.1105/tpc.112.102608 PMC3517241

[34] Jung J. , Yeom E., Choi K.W. Ciao1 interacts with crumbs and Xpd to regulate organ growth in *Drosophila*. Cell Death Dis. 2020; 11:365. 32404863 10.1038/s41419-020-2564-3 PMC7220951

[35] Maeda I. , Kohara Y., Yamamoto M., Sugimoto A. Large-scale analysis of gene function in *Caenorhabditis elegans* by high-throughput RNAi. Curr. Biol. 2001; 11:171–176. 11231151 10.1016/s0960-9822(01)00052-5

[36] Lombaerts M. , Tijsterman M., Verhage R.A., Brouwer J. *Saccharomyces cerevisiae* mms19 mutants are deficient in transcription-coupled and global nucleotide excision repair. Nucleic Acids Res. 1997; 25:3974–3979. 9321645 10.1093/nar/25.20.3974 PMC147023

[37] Wang Y. , Singh R., Tong E., Tang M., Zheng L., Fang H., Li R., Guo L., Song J., Srinivasan R. et al. Positional cloning and characterization of the papaya diminutive mutant reveal a truncating mutation in the CpMMS19 gene. New Phytol. 2020; 225:2006–2021. 31733154 10.1111/nph.16325

