# Supplementary material for: The critical role of the iron–sulfur cluster and CTC components in DOG-1/BRIP1 function in Caenorhabditis elegans
Source: Nucleic Acids Res. 2024 Jul 16;52(16):9586–95. doi: 10.1093/nar/gkae617 (PMC11381322; doi:10.1093/nar/gkae617)
Supplement: gkae617_Supplemental_File [file gkae617_supplemental_file.pdf]

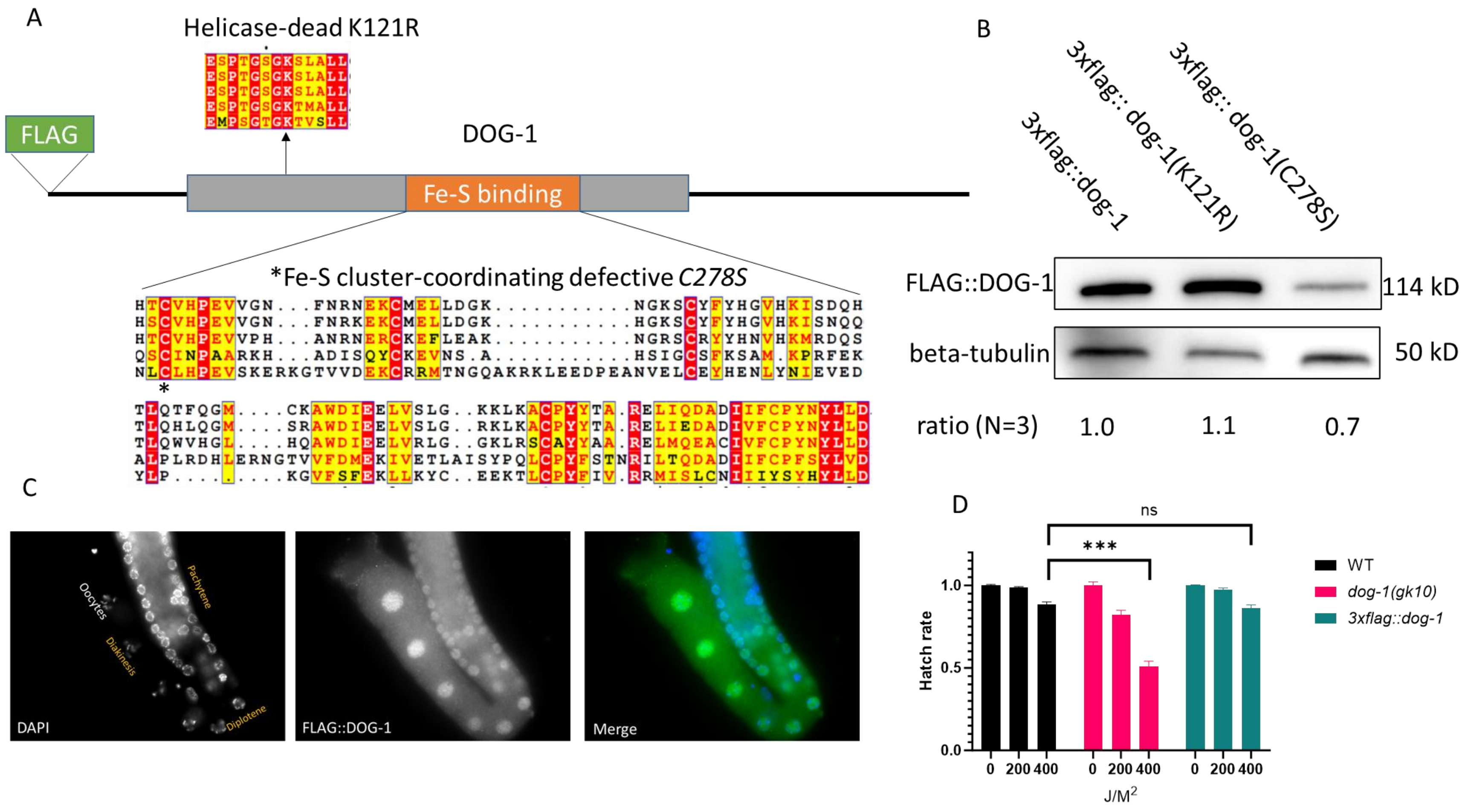

Figure S1 (A) Schematic representation of iron-sulfur binding defective DOG-1 (C278S), helicase-dead DOG-1(K121R) and FLAG::DOG-1. Protein sequences used for alignment are gi|301897118|ref|NP_114432.2| Fanconi anemia group J protein [*Homo sapiens*], gi|30795235|ref|NP_840094.1| Fanconi anemia group J protein homolog [*Mus musculus*], gi|160333450|ref|NP_001103766.1| brip1 gene product [*Danio rerio*], >NP_493618.1 Helicase ATP-binding domain-containing protein [*Caenorhabditis elegans*], and RAD3. (B) Reduced DOG-1(C278S) protein level in total worm lysate. (C) Nuclear localization of FLAG::DOG-1 in *C. elegans* germ cells. (D) *3xflag::dog-1* worms are not sensitive to UVA/TMP-induced ICLs.

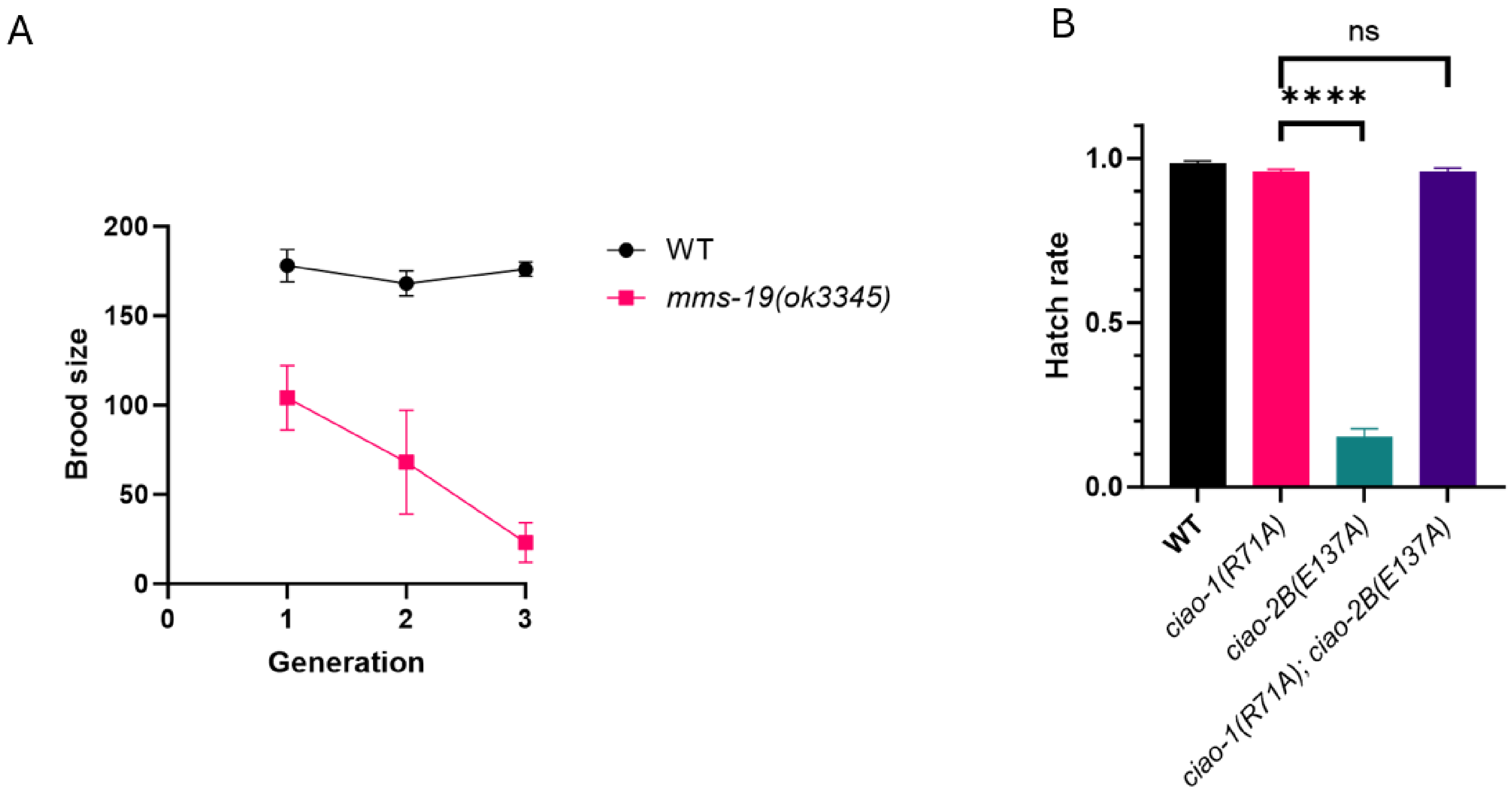

Figure S2 (A) *mms-19(ok3345)* brood size reduces over three generations at 25°C. (B) The hatch rate of *ciao-2B(E137A)* is reduced at 25°C indicating *E137A* is a temperature-sensitive allele. This lethality is rescued by the addition of *ciao-1(R71A)* as seen in the double mutant.

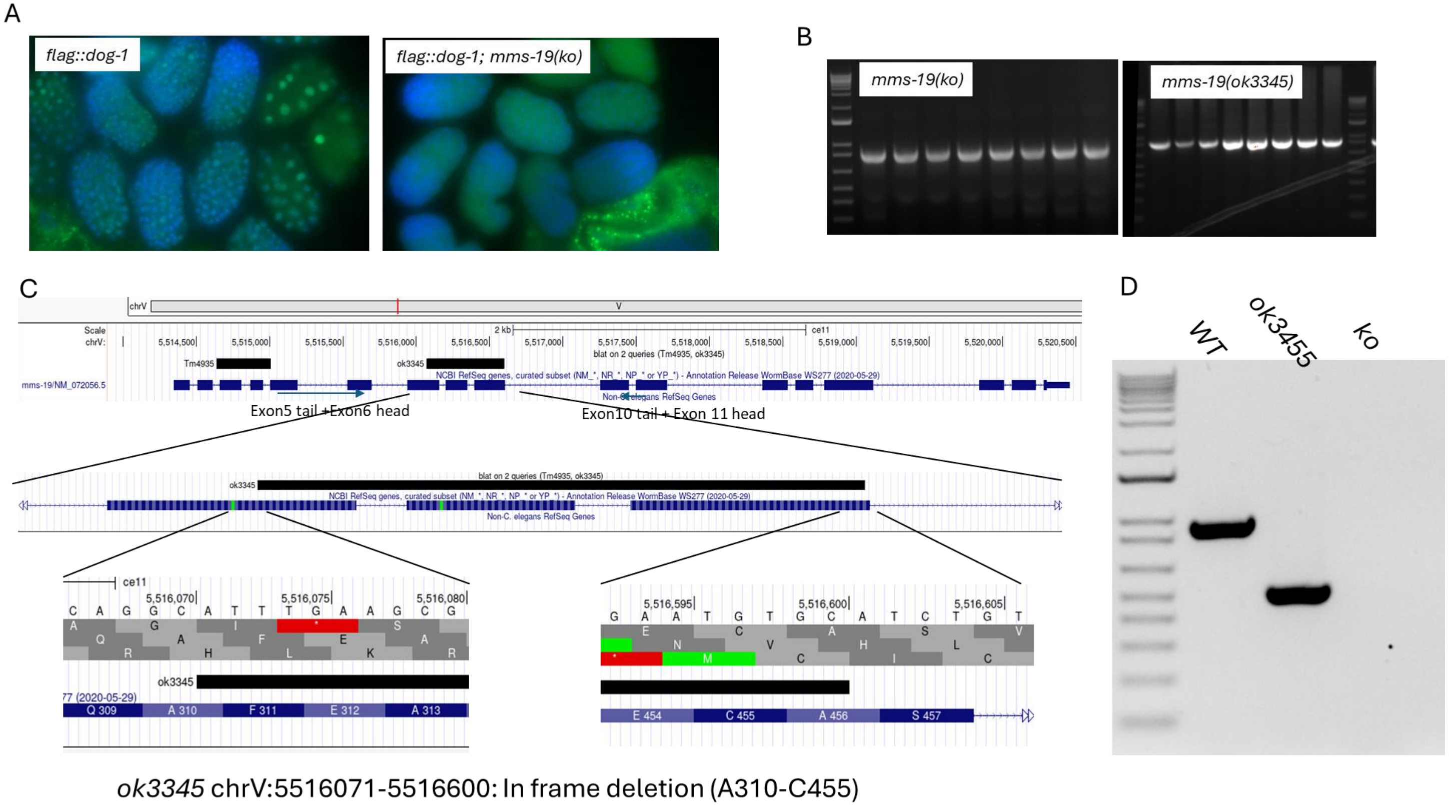

Figure S3 (A) FLAG::DOG-1 fails to localize in the nucleus in *mms-19(ko)* embryos. (B) No elevated guanine-rich DNA deletion is observed in either *mms-19(ko)* or *mms-19(ok3345)* worms (C) *mms-19(ok3345)* deleting partial exon 7, 8 and partial exon 9 is expected to produce an in-frame deletion (A310 –C455) (D) RT-PCR verification of *ok3345* in-frame deletion. Primers that span from junctions of exon 5/6 (oMTG598 GAGTTTAAGCCGCAAAAAACGG) and 10/11 (oMTG599 ACTTTCATTGCGTTCCAAGAATTAGT) were used to amplify the *ok3345* locus from total RNA extracts of N2, *mms-19(ok3345)* and *mms-19(ko).* A band of 952 bp for wild-type transcript and a band of 515 bp for *ok3345* deletion were observed on the 2% agarose gel. PCR products sequence were confirmed by Sanger sequencing.

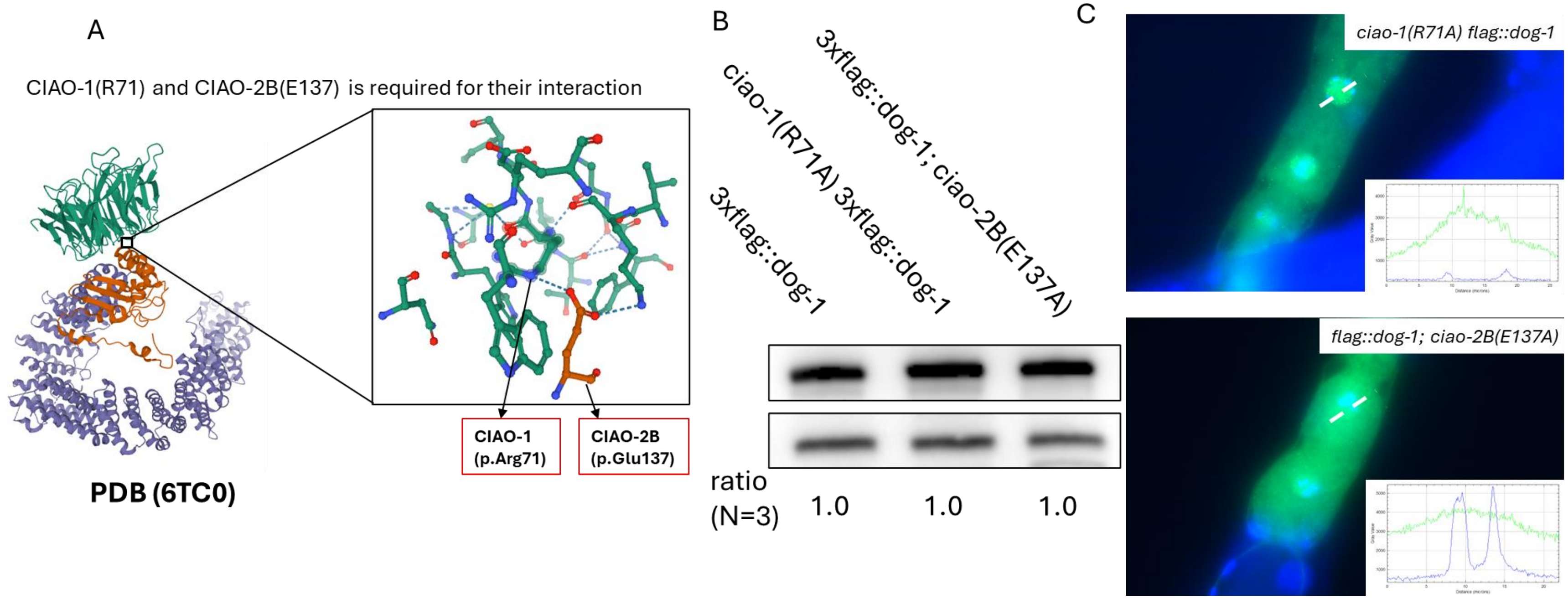

Figure S4 (A) Crystal structure of MMS19-CIAO1-CIAO2B CIA targeting complex. CIAO-1(R71) and CIAO-2B(E137) are at the interaction interface (structure from PDB(6TC0)). (B) Immunoblot of total worm lysates indicates unchanged protein level of FLAG::DOG-1 in *ciao-1(R71A)* and *ciao-2B(E137A)* worms. (C) Immunostaining of oocytes indicates nuclear localization of FLAG::DOG-1 in *ciao-1(R71A)* and *ciao-2B(E137A)* worms. Line scan analysis across an oocyte reveals the nucleocytoplasmic distribution of FLAG::DOG-1 (green) relative to the nucleus (DAPI).

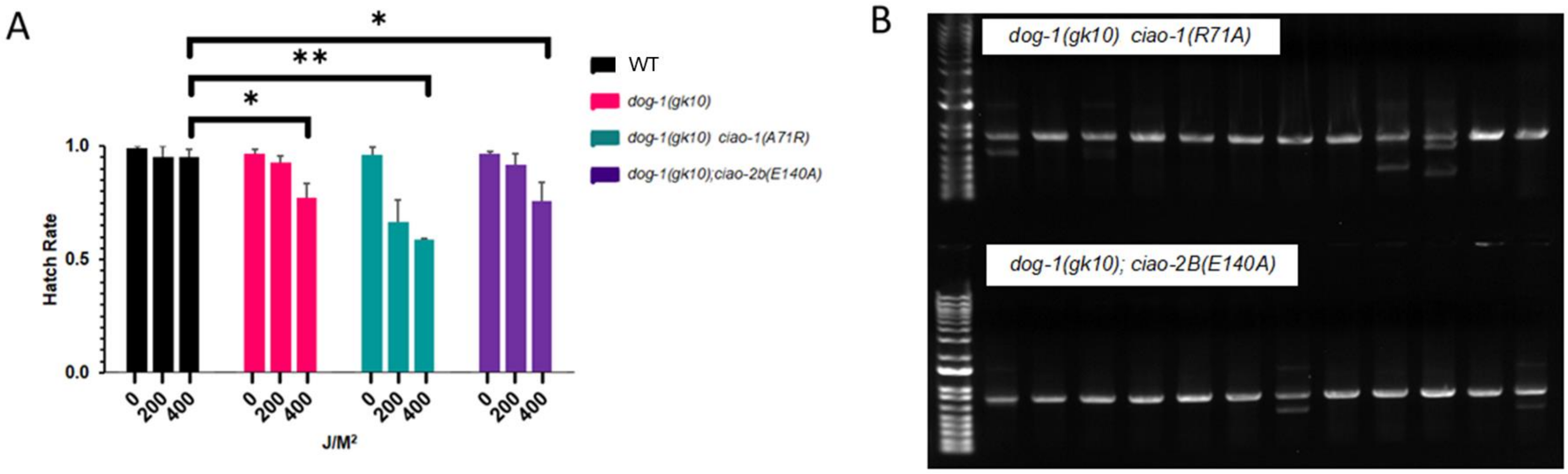

Figure S5 (A) Double mutant *dog-1(gk10) ciao-1(R71A)* and *dog-1(gk10); ciao-2B(E137A)* sensitivity to TMP/UVA-induced ICLs. Error bars represent the standard error of the mean. (B) G-rich DNA deletion is observed in *dog-1(gk10) ciao-1(R71A)* and *dog-1(gk10); ciao-2B(E137A)*.

Table S1 G-4 DNA-induced deletion at *qua830* locus by single worm PCR

Genotype	*Number of single worm PCR*	*G-tract deletion*	*Percentage %*	*p-value (comparison with WT)*	*p-value (comparison with dog-1(gk10)*
WT	*138*	*0*	*0*	NA	NA
dog-1(gk10)	*151*	*53*	*35*	4.95e-16	NA
ciao-1(ko)	*26*	*0*	*0*	NA	
ciao-2B(ko)	*28*	*0*	*0*	NA	
mms-19 (ko)	*128*	*0*	*0*	NA	
mms-19(ok3345)	*138*	*0*	*0*	NA	
ciao-1(R71A)	*210*	*2*	*1*	0.252	4.08e-21
ciao-2B(E137A)	*206*	*1*	*0.5*	0.414	1.04e-21
ciao-1(R71A); ciao-2B(E137A)	*228*	*0*	*0*	NA	
dog-1(K121R)	*30*	*13*	*43*	2.65e-19	0.395
dog-1(C278S)	*32*	*15*	*47*	2.65e-19	0.213
dog-1(gk10) ciao-1(R71A)	*59*	*19*	*32*	1.86e-21	0.693
dog-1(gk10); ciao-2B(E137A)	*60*	*16*	*27*	7.74e-14	0.241

Note: a two-sample t-test was used to compare the frequency of G-tract deletions between various genotypes and the control group (*WT*), as well as the positive control (*dog-1(gk10)*).

Table S2 Phenotypic outcomes in the absence of CTC Components

H. sapiens	*C. elegans*	Alleles	Mutation type	Phenotype
CIAO1	*Y18D10A.9/ciao-1*	*gk5013 (ko)*	CRISPR Knockout CDS deletion	Lethal (sterile)
		R71A	Human p.Arg65Ala mimic	Viable
CIAO2B	*F45G2.10/ciao-2B*	*gk5482(ko)*	CRISPR knockout CDS deletion	Lethal (sterile)
		E137A	Human p.Glu140Ala mimic	Viable (ts)
		E137D	Human p.Glu140Asp mimic	Viable
MMS19	*mms-19*	*ok3345*	In-frame deletion from p.Ala310 to Cys455	Viable (mrt)
		mod412 (ko)	CRISPR knockout CDS deletion	Viable
BIRP1	*dog-1*	*gk10 (ko)*	Deletion/null	Viable

Table S3 Alleles used in this study

Genotype	*Full genotype*	*Notes*	MTG strains #
WT	*wildtype*	CGC1 (formerly known as PD1074)	MTG274
ciao-1(ko)/+	*Y18D10A.9(gk5013[loxP + myo-2p::GFP::unc-54 3' UTR + rps-27p::neoR::unc-54 3' UTR + loxP])/+ I*	*C. elegans Gene Knockout Lab*	MTG10
ciao-2B(ko)/+	*F45G2.10(gk5482[loxP + myo-2p::GFP::unc-54 3' UTR + rps-27p::neoR::unc-54 3' UTR + loxP])/+ II*	*C. elegans Gene Knockout Lab*	MTG207
mms-19(ko)	*mms-19(mod412)*	*CRISPR allele, This study*	MTG574
mms-19(ok3345)	*C24G6.3(ok3345)*	*International C. elegans Gene Knockout Consortium*	MTG14
ciao-1(R71A)	*ciao-1(mod409[R71A])*	*CRISPR allele, This study*	MTG571
ciao-2B(E137A)	*ciao-2B(mod334[E137A])*	*CRISPR allele, This study*	MTG479
dog-1(gk10)	*dog-1(gk10) I*	*International C. elegans Gene Knockout Consortium*	MTG184
dog-1(C278S)	*dog-1(mod461[dog-1(C278S)])*	*CRISPR allele, This study*	MTG633
dog-1(K121R)	*dog-1(mod463[dog-1(K121R)])*	*CRISPR allele, This study*	MTG635
dog-1::flag	*dog-1(mod218[dog-1::3xflag])*	*CRISPR allele, This study*	MTG301
dog-1::flag(C278S)	*dog-1(mod462[dog-1(C278S)::3Xflag])*	*CRISPR allele, This study*	MTG634
dog-1::flag(K121R)	*dog-1(mod464[dog-1[K121R]::3Xflag])*	*CRISPR allele, This study*	MTG636
dog-1(gk10) ciao-1(R71A)	*dog-1(gk10) ciao-1(mod409[R71A]) I*	*CRISPR allele, This study*	MTG783
dog-1(gk10);ciao-2B(E137A)	*dog-1(gk10); ciao-2B(mod334[E137A])*	*CRISPR allele, This study*	MTG784
ciao-1(R71A); ciao-2b(E137A)	*ciao-1(mod409[R71A]); ciao-2B(mod334[E137A])*	*CRISPR allele, This study*	MTG609

Table S4 Guide RNAs and single-strand DNA repair templates

Mutation	crRNA	*ssODN*
mms-19(ko)	ATTAAGTTTCAAAGAAGGAA(N);<br>CTAGTTTGTCGTTAATAACC (C)	*TCGCAACTTAATTCTTCGATAACGAAGCACCTTTCTATTAACGACAAACTAGATATACTTTTGTTTTGTCCTAAATTGT*
ciao-1(R71A)	GGATGACAGTCATACACGGG	*CAAAACTCGCAGAAACTAGACATTTTCCGTCATTTGAAAATGCGACTGAGGCAACAGCCCGTGTATGACTGTCATCCAGTGTTGTACGACACTCCAGACGCA*
ciao-2B(E137A)	GGAACGAGTAGCTGCTGCAA	*GCCCCTCAAAAAGTTAATTTTTAAAACATAAATTCAAAATGTCTCATTTAGGACCGAGTAGCTGCTGCAATGGAAAATCAAGGACTAATGCACGCTGTTAACGAATGCCTCAGAGTTCCAGAA*
dog-1(C278S)	GATGTCTGCGTGTTTACGAG	*TTGTATTCAGACACACAATTCTCGCAAGCAGAGAACAATCTAGTATCAACCCTGCTGCTCGTAAACACGCAGACATCTCACAATACTGTAAAGAAGTGAAC*
dog-1(K121R)	GACGGGAAGTGGCAAAACTA	*CGGCGCTGAAAAATAGTCAAAATGTGCTTGGCGAGTCGCCGACGGGAAGTGGCCGCACTATGGCTCTGCTGGCATCTACGTGTGCATGGCTCAAGCAGTATAT*
dog-1::flag	TCTCCAAAATGCATCGCTTG	*GTTTTCGTTATTGCGATTTATAGAAAAATACCATTTGCAGGTAATTATGGATTACAAAGACCATGATGGTGACTATAAGGATCATGATATTGACTATAAAGACGATGACGATAAGTCATCAAGCGATGCATTTTGGAGAATGTTCGCAAACAAAAACAAAGGAAAATCGAATACCCGATCGGCATTTCA*
